# Quantum Memristors with Superconducting Circuits

**DOI:** 10.1038/srep42044

**Published:** 2017-02-14

**Authors:** J. Salmilehto, F. Deppe, M. Di Ventra, M. Sanz, E. Solano

**Affiliations:** 1 Department of Physics, Yale University, New Haven, Connecticut 06520, USA; 2 Department of Physical Chemistry, University of the Basque Country UPV/EHU, Apartado 644, E-48080 Bilbao, Spain; 3 QCD Labs, COMP Centre of Excellence, Department of Applied Physics, Aalto University, P.O. Box 13500, FI-00076 Aalto, Finland; 4 Walther-Meißner-Institut, Bayerische Akademie der Wissenschaften, D-85748 Garching, Germany; 5 Physik-Department, Technische Universität München, D-85748 Garching, Germany; 6 Nanosystems Initiative Munich (NIM), Schellingstraße 4, 80799 München, Germany; 7 Department of Physics, University of California, San Diego, La Jolla, CA 92093, USA; 8 IKERBASQUE, Basque Foundation for Science, Maria Diaz de Haro 3, 48013 Bilbao, Spain

## Abstract

Memristors are resistive elements retaining information of their past dynamics. They have garnered substantial interest due to their potential for representing a paradigm change in electronics, information processing and unconventional computing. Given the advent of quantum technologies, a design for a quantum memristor with superconducting circuits may be envisaged. Along these lines, we introduce such a quantum device whose memristive behavior arises from quasiparticle-induced tunneling when supercurrents are cancelled. For realistic parameters, we find that the relevant hysteretic behavior may be observed using current state-of-the-art measurements of the phase-driven tunneling current. Finally, we develop suitable methods to quantify memory retention in the system.

Circuit elements that intrinsically carry a recollection of their past evolution^1^,^2^,^3^ promise to bring forth novel architectural solutions in information processing and unconventional computing^4^ due to their passive storage capabilities. These history-dependent circuit elements can be both dissipative and non-dissipative, such as memcapacitors and meminductors^2^,^5^, or just dissipative, such as memristors. Classical memristors^6^,^7^,^8^,^9^ are elements whose operational definition relates the voltage *V* and the current *I*, complemented with an update of one or more internal variables *x* carrying information of the electrical history of the system. For a voltage-controlled memristor


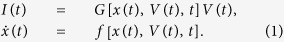


The memductance (memory conductance) *G* depends on both the instantaneous input voltage *V* and the state variable *x*, which tracks the past memristor configuration via the update function *f*. Such dynamics leads to the characteristic pinched hysteresis loops under periodic driving^3^,^6^,^7^,^10^,^11^, a strictly non-linear conductive effect showcasing zero-energy information storage^1^.

Even though both the quantization of superconducting circuits^12^ and applications of memristors are well established techniques, memristive operation in the realm of quantum dynamics is a largely unexplored area. From an intuitive point of view, the combination of powerful memristive concepts with quantum resources, such as superposition and entanglement, promises groundbreaking advances in information and communication sciences. With this motivation in mind, the idea of a quantum memristor was recently defined in ref. 13 by introducing the fundamental components for engineering memristive behavior in quantum systems. However, superconducting circuits naturally include memristive elements in Josephson junctions, a feature exploited in a recently proposed classical superconducting memristor design^14^. While this conductance asymmetric superconducting quantum interference device (CA-SQUID) design was able to produce hysteretic behavior^14^, it did not include the quantum features of the circuit, including the dissipative origins of the memory or its measurement and quantification. These features are of utmost importance, as the operation of the design is based on quasiparticle tunneling, whose control and measurement have recently seen significant strives forward^15^,^16^. Indeed, to our knowledge, up to now no experimental work has studied the hysteretic IV-characteristics of such systems. In our opinion, the reasons for this are two-fold, namely, 1) the pinched hysteresis loops were only recently predicted to exist for such systems in ref. 14 with the use of the aforementioned CA-SQUID and a proper selection of parameters, and 2) the experimental apparatus required to control and measure quasiparticle excitations with high accuracy is just beginning to emerge (see refs 15 and 16).

In this Article, we show that a suitably designed superconducting quantum circuit element with an external phase bias serves as a prototypical quantum memristor via low-energy quasiparticle tunneling. To this end, we describe the device in a fully quantum-mechanical fashion. We apply an ensemble interpretation of the system input and output, while the average superconducting phase difference stores information of the past dynamics. We study the hysteretic signature in a regime achievable with recent quantum nondemolition projective measurements^16^, and construct a memory quantifier related to the accumulation of internal state change. Finally, we discuss the quantumness of our proposal, comparing it with ref. 14. Our proposal represents, to our knowledge, the first design of a superconducting quantum memristor from fundamental principles, exploiting quasiparticle tunneling in memristive quantum information processing.

The envisioned device has the rf SQUID design shown in Fig. 1(a). It consists of a superconducting loop with inductance *L*, which is interrupted by a dc SQUID with negligible loop inductance acting as an effective flux-tunable Josephson junction. The dc SQUID junctions are made from different materials so that they have the same critical current but a different normal conductance^14^. In this way, the effective critical current of the dc SQUID can be completely suppressed by a bias flux of half a flux quantum, Φ_0_/2, threading its loop^14^. Finally, we also apply a bias flux Φ_*d*_ to the rf SQUID loop, resulting in the phase bias 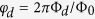.

The total Hamiltonian of this device is the sum of the system Hamiltonian 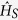, a term for the quasiparticle degree of freedom, and a total tunneling term. The latter includes quasiparticle contributions but, due to the vanishing effective critical current (note that these contributions would yield a renormalization of the qubit frequency in the low-energy regime considered in ref. 17), neither pair contributions, nor the Josephson counterterm^17^,^18^,^19^. Under these conditions, the system Hamiltonian takes the harmonic form


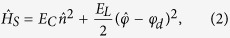


where 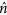 and 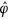 are the Cooper-pair counting and phase difference operators of the effective junction, respectively. We define the capacitive energy scale 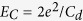 with the intrinsic junction capacitance *C*_*d*_ and the inductive energy scale *E*_*L*_ = (1/*L*) (Φ_0_/(2*π*))^2^. Regarding the dc SQUID, we assume the limit of strong conductance asymmetry needed for the effective junction picture due to the inclusion of quasiparticle excitations (see Supplementary Information). In this limit, the dissipative flow is through the physical junction with a smaller superconducting gap while the junction with a larger gap functions as a shunt for the total Josephson current through the SQUID. Furthermore, we demand that the phase bias is changed adiabatically, i.e., sufficiently slowly to avoid the generation of quasiparticles. Finally, our device operates in the low-energy regime 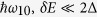, where 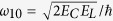 is the system transition frequency and *δE* is the characteristic energy of the quasiparticles above the gap Δ. Even though the Hamiltonian does not warrant operation as a qubit due to the lack of sufficient anharmonicity, the system dynamics is confined to the two lowest eigenstates of Eq. (2) when the aforementioned assumptions are complemented with operation in the high-frequency regime 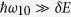. In this regime, there exist no quasiparticles with sufficiently high energy to excite the system. We emphasize that the slow biasing and high frequency assumptions utilized in this article are not contradictory. The former refers to suppressing unwanted generation of quasiparticles due to the biasing field^20^ while the latter refers to a condition on the quasiparticle bath.

The two-level master equation describing the quasiparticle-induced decay takes the Lindblad form^19^ 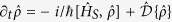 for the system density 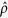, with 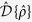 the corresponding Lindbladian dissipator. Note that the master equation assumes adiabatic steering, and employs the Born-Markov and secular approximations. We omit the quasiparticle-induced average frequency shift and the pure dephasing channel. See Supplemental Material for the estimation of these effects. In the low-energy limit, the decay rate factorizes into


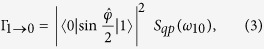


in the lowest order in *ω*_10_/Δ. Here, {|0〉, |1〉} are the lowest energy eigenstates of 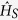 and the quasiparticle spectral density *S*_*qp*_(*ω*) now depends on the distribution function which may, in general, include both equilibrium and non-equilibrium contributions. Note that the decay rate in Eq. (3) stems from the 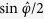 dependence of the quasiparticle–system coupling and is crucial to the memristive behavior detailed in the following section. By using the properties of displaced number states (see Supplementary Information), the squared inner products in Eq. (3) have a convenient cosine form valid for any pair of Fock states {|*n*〉, |*m*〉},


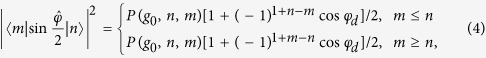


with


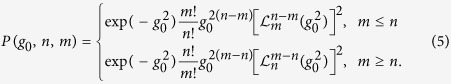


Here, *g*_0_ = [*E*_*C*_/(32*E*_*L*_)]^1/4^ and 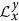 denotes an associated Laguerre polynomial. Notably, the sign of the cosine term in Eq. (4) depends on the parity difference between the states involved. While this potentially provides insight into interesting phenomena when multiple decay channels are involved^21^,^22^, we concentrate on the two-level process and leave such considerations for future studies.

To understand how memristive behavior emerges from quasiparticle tunneling, we study the charge flow in the device. Let 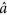 be the annihilation operator for a harmonic excitation in the system. This allows us to write 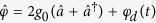 and 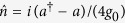, and denote by 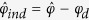 the operator for the phase over the rf SQUID loop inductance. The directional convention for the superconducting phase differences and the different currents are presented in Fig. 1(b). The average charging current 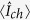 and the inductive current 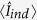 can be rigorously derived (see Supplementary Information) to obtain, by current conservation, the average quasiparticle current through the effective junction. The result is 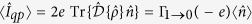, which corresponds to the dissipative current induced by the interaction with the quasiparticle bath represented by the dissipator 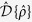. Using 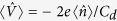, the average quasiparticle current is determined by


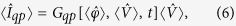


where we have preemptively written the effective conductance as a function of the selected memory variable 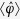, input  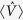, and time *t*. Solving for the dynamics, we obtain


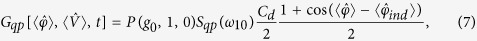


where the average inductive phase difference only requires knowledge of the input via


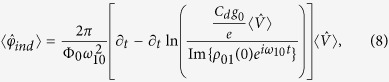


and we denoted the initial system coherence in the energy eigenbasis by 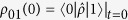. The memory variable update function in 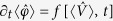 only depends on the input and time, and has the explicit form


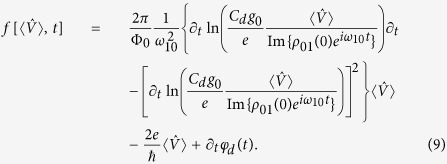


Equations (6),(7),(8),(9) indicate that a simple superconducting device operates as a voltage-controlled quantum memristor when the average voltage over a tunneling element is interpreted as the system input, the average quasiparticle tunneling current as the output, and the average superconducting phase difference as the memory retention variable. The quasiparticle conductance acts as the memductance corresponding to the memory-dependent average current response. It should be noted that physically speaking our device is considered a flux-controlled memristive device as it includes non-zero capacitive and inductive elements^2^ while having no external capacitive coupling. However, only considering the quasiparticle contribution to the current and studying the above-mentioned equations allows us to define the device as a voltage-controlled memristor from an operational point-of-view.

The operation of the constructed memristor is of ensemble nature, that is, the system input and output are quantum averages obtained from the measurement record of the corresponding observables. Experimental input consists of initialization and a slowly oscillating flux bias applied to the rf SQUID loop. In this way, one obtains independently generated records which, consequently, have a complex correlation exhibiting memory features via Eq. (6). In fact, the selected system input is not independent of the decay, but experiences a memory-dependent damping


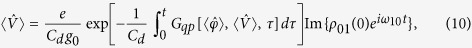


which allows one to self-consistently solve the fundamental equations above. One such solution is identifiable as mimicking the operation of the classical superconducting memristor^14^, in which the memory is fully stored in the phase bias. It is obtained in the weak-damping limit by initializing the system with 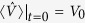 and 
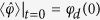
, and by assuming a resonant sinusoidal phase bias 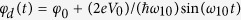. Weak voltage damping implies that 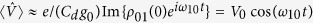, where the update is given by the classical Josephson relation 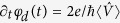. The solution embodies the two implicit assumptions for the classical memristor: (1) the rf SQUID loop has a negligible inductance, and (2) the internal dynamics is negligibly affected by the same dissipation that produces the output.

As a first step, we need to verify whether the above-described classical-limit solution is consistent with the semiclassical results of ref. 14. In Fig. 2, one clearly sees that we observe the hysteretic current-voltage characteristic curves as required for a memristive element. In other words, a proper choice of the sinusoidal drive allows for tunable finite-area pinched loops^3^. Employing the system parameters from Fig. 2, the above weak-damping solution is accurately numerically retrieved with *S*_*qp*_(*ω*_10_) = 10^−4^*ω*_10_ over multiple oscillation periods. This corresponds to a minimum relaxation time during the driving period of min(*T*_1_) ∝ 1 *μ*s relevant to the current state-of-the-art experimental setups^15^,^16^. While those setups consider a different type of system, the fluxonium, very little experimental work has been able to reach the regime in which quasiparticle-induced relaxation is observable and, consequently, we use these references for initial comparison. Even though 
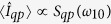
, the magnitude-scaled hysteresis curve is robust against decreasing the minimum *T*_1_-time by 2 orders of magnitude (see Supplementary Information). Beyond this, the input and output values are subject to noticeable decay. We show the parametric dependence of the average voltage and quasiparticle current in Fig. 2 for *S*_*qp*_(*ω*_10_) = *ω*_10_ corresponding to min(*T*_1_) ∝ 100 ps. The hysteresis curve starts from a point in the weak-damping trajectory due to the identical initialization, and it is followed by a reduction of the area with time. The time evolution in Fig. 2 shows a gradual decay in the voltage and current amplitudes. Note that the system is operated in the phase regime of almost negligible loop inductance. This allows for a feasible resonant phase biasing frequency *ω*_10_/2*π* ≈ 45 GHz, achieved while ensuring sufficient adiabaticity max(*α*_*rs*_) ≈ 0.15 (see Supplementary Information), necessary for the master-equation treatment employed for the quasiparticle bath.

The initialization of the system plays a crucial role in the operation and does not simply determine the initial position in the parametric curve. Figure 3 shows the hysteresis curves for three different initializations, assuming the weak-damping limit and a resonant sinusoidal drive protocol. These curves can be interpreted by studying the time symmetry of the quasiparticle current between two consecutive crossings of the zero-energy point and indicate a tunable landscape of hysteretic behavior (see Supplementary Information).

To quantify the non-Markovian^23^ character of the device, we consider the area enclosed by a hysteresis loop in the current-voltage plane as a memory measurement. This interpretation is founded in the observation that the absence of area correlates with purely time-local current response. In other words, a nonlinear conductance cannot produce a non-zero area since it depends only on the instantaneous value of the voltage. The memory quantifier for the *k*th traversed loop takes the form 
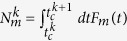
 (See Supplementary Information), where 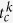 fulfills 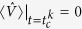 for each *k*. This quantifier stores the evolution information of 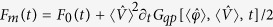, where


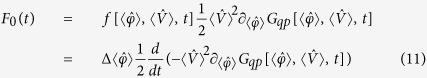


corresponds to the response specific to the selected memory variable, and the second term to the explicit time dependence of the memductance not included in the internal memory variable. However, it is in principle always possible to redefine the memory variable to absorb the explicit time dependence in the memductance, so that *F*_*m*_(*t*) = *F*_0_(*t*). The two expressions in Eq. (11) imply that the quantifier corresponds to a time-dependent weighted record of the change in the memory variable 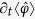 or the instantaneous distance from its initial value 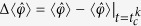. If the conductance is a non-linear function of only the instantaneous input, *F*_*m*_(*t*) vanishes in integration due to input periodicity. See Supplemental Information for the decay of the quantifier as well as its response to different initializations.

Finally, our quantum memristor is formulated in the ideal case of zero leakage supercurrent. Adding a nonzero pair-tunnelling term, not only modifies the energy and state structure, but inflicts a Josephson tunneling current which may disrupt the operation. While there can be multiple factors contributing to the leakage supercurrent, such as magnetic flux noise, the primary experimental factor to tackle is possibly the critical current imbalance in the SQUID. The state-of-the-art critical current suppression factor based purely on fabrication techniques is ~10^−2^ while the balanced SQUID^24^ promises a factor of ~10^−3^–10^−4^, for a maximum critical current of 30 nA. In terms of the Hamiltonian, this implies that the imbalance term is 10^−1^–10^−3^ times the charging energy scale used here. In addition, our formulation assumes only the quasiparticle decay channel and omits other natural loss channels (dielectric, inductive, radiative). Recent experimental work has studied quasiparticle-limited relaxation and shown significant progress in suppressing the additional decay channels, modifying the quasiparticle population through different means, and discerning between the different decay mechanisms^15^,^16^.

Let us finish with a brief discussion about the quantumness of the system, as well as the role of superposition and entanglement. The dynamics of the quantum memristor described above is purely quantum, in the strict sense that the evolution cannot be emulated by a classical channel^25^,^26^. This is not surprising, since the quantumness of our design refers to the full dissipative treatment (as an open quantum system) of the quasiparticle bath leading to memristive features in the expectation values of quantum observables. Therefore, superposition plays the same role as in any other quantum system. With respect to the entanglement, coupling two of these quantum memristors is a natural and relevant question after showing the dynamics of a single device, but beyond the scope of this manuscript.

In conclusion, we have demonstrated a prototype design for a quantum memristor in a superconducting circuit relying on quasiparticle tunneling. The pinched hysteretic behavior of the average quasiparticle current is a clear signature of conductance beyond typical non-linearity, and modified by both the characteristics of the circuit and the quasiparticle bath. The measurement resolution can potentially be varied by tuning the non-equilibrium quasiparticle population, by just using the state-of-the-art injection and trapping methods^16^ during the lifetime of the quasiparticles. Our work paves the way for the engineering of on-demand quantum non-Markovianity using the superconducting quantum memristor as a building block. Furthermore, we may consider possible applications such as the codification of quantum machine learning protocols^27^,^28^ and neuromorphic quantum computing^13^.

## Additional Information

**How to cite this article:** Salmilehto, J. *et al*. Quantum Memristors with Superconducting Circuits. *Sci. Rep.* **7**, 42044; doi: 10.1038/srep42044 (2017).

**Publisher's note:** Springer Nature remains neutral with regard to jurisdictional claims in published maps and institutional affiliations.

## Supplementary Material

Supplementary Information

## Figures and Tables

**Figure 1:**
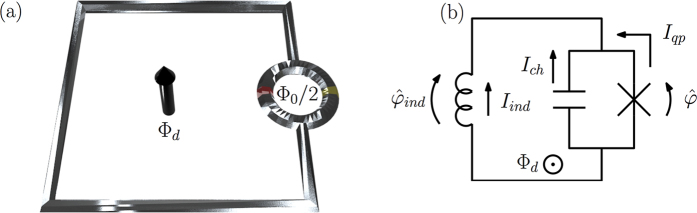
Superconducting quantum memristor. (**a**) Schematic representation of the superconducting quantum memristor. The green and red strips represent junctions with different normal conductances. (**b**) Current diagram using effective circuit elements corresponding to the total loop inductance, charge retention of the SQUID, and the quasiparticle tunneling through it. Let us remark that, as the capacitative part has been explicitly separated in (**b**), the cross-notation does not refer here to the entire (effective) Josephson junction, but to the quasiparticle and phase-dependent dissipative current contributions.

**Figure 2:**
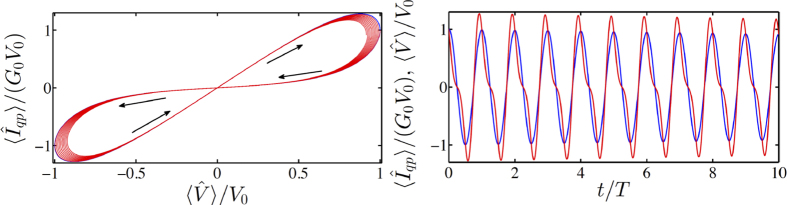
Left panel: Parametric hysteresis curve (red) and the weak-damping solution (blue). Right panel: Temporal evolution of average voltage (blue) and quasiparticle current (red). We use initialization 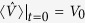, 
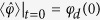
 and resonant sinusoidal phase bias. Parameters are *E*_*C*_/(2*πħ*) = 1 GHz, *E*_*L*_ = 10^3^*E*_*C*_, *φ*_0_ = *π*/2, *S*_*qp*_(*ω*_10_) = *ω*_10_, and 2*eV*_0_/(*ħω*_10_) = 1. The arrows indicate the direction of temporal evolution recorded over 10 oscillation periods *T* = 2*π/ω*_10_ and we use the shorthand notation *G*_0_ = *P(g*_0_, 1, 0) *S*_*qp*_(*ω*_10_)*C*_*d*_/4.

**Figure 3:**
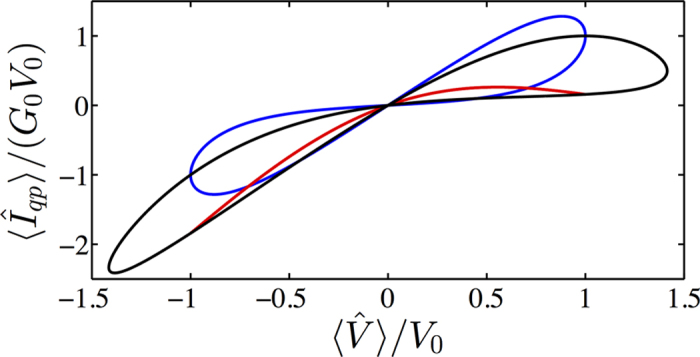
Hysteresis curves with resonant sinusoidal phase bias in the weak-damping limit. System initialized such that 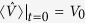, 
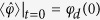
 (blue), 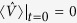, 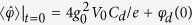 (red), and 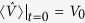, 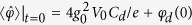 (black). The system parameters are the same as in Fig. 2 with *S*_*qp*_(*ω*_10_) = 10^−4^*ω*_10_.
