# Supplementary material for: Quantum Memristors with Superconducting Circuits
Source: Sci Rep. 2017 Feb 14;7:42044. doi: 10.1038/srep42044 (PMC5307327; doi:10.1038/srep42044)
Supplement: Supplementary Information [file srep42044-s1.pdf]

Supplemental Material for Quantum Memristors with Superconducting Circuits

J. Salmilehto,[1,2,3] F. Deppe,[4,5,6] M. Di Ventra,[7] M. Sanz,[2] and E. Solano[2,8]

[1]*Department of Physics, Yale University, New Haven, Connecticut 06520, USA*
[2]*Department of Physical Chemistry, University of the Basque Country UPV/EHU, Apartado 644, E-48080 Bilbao, Spain*
[3]*QCD Labs, COMP Centre of Excellence, Department of Applied Physics, Aalto University, P.O. Box 13500, FI-00076 Aalto, Finland*
[4]*Walther-Meißner-Institut, Bayerische Akademie der Wissenschaften, D-85748 Garching, Germany*
[5]*Physik-Department, Technische Universität München, D-85748 Garching, Germany*
[6]*Nanosystems Initiative Munich (NIM), Schellingstraße 4, 80799 München, Germany*
[7]*Department of Physics, University of California, San Diego, La Jolla, CA 92093, USA*
[8]*IKERBASQUE, Basque Foundation for Science, Maria Diaz de Haro 3, 48013 Bilbao, Spain*

THE EFFECTIVE JUNCTION PICTURE IN THE PRESENCE OF QUASIPARTICLE EXCITATIONS

While it is well-known that a SQUID behaves as if it was a single effective junction with a tunable Josephson energy when pair tunneling is discussed, additional conductance requirements are set by the inclusion of quasiparticle excitations. Classically speaking, a non-vanishing phase-dependent conductance can be achieved in a conductance-asymmetric SQUID, while assuming that the physical junctions have equivalent critical currents [1]. Summation of dissipative currents yields a representation as an effective junction with an effective leakage conductance $G_{\text{eff}} = G_1 + G_2$, where G_i is the leakage conductance of the ith physical junction, and an asymmetry term for the phase-dependent current of $S_{\text{asym}} = (G_1 - G_2)/(G_1 + G_2)$ [1].

To use the effective junction picture in the main text in association with quasiparticle tunneling, we assume strong asymmetry $G_1 \gg G_2$ such that $S_{\text{asym}} \approx 1$. Hence, the dissipative flow is effectively only through a single junction, while the total pair current can still be cancelled. This allows for the effective junction picture to be used for describing quasiparticle tunneling. Since the Ambegaokar-Baratoff relation implies for equivalent critical currents that $G_1/G_2 = \Delta_2/\Delta_1$, with Δ_i is the superconducting gap of the electrodes of the ith physical junction of the SQUID, this assumption can be enforced by demanding $\Delta_2 \gg \Delta_1$. Weakening the assumption would require the degrees of freedom of the individual physical qubits to be included in the Hamiltonian in Eq. (2) of the manuscript. This means that the quasiparticle tunneling Hamiltonian would have to be given for each physical junction separately, resulting in two separate decay channels, which allows for the weaker conductance discrepancy to manifest. We leave such considerations for future work and assume the limit of strong conductance asymmetry throughout the main text.

ESTIMATION OF ADIABATICITY, QUASIPARTICLE-INDUCED AVERAGE FREQUENCY SHIFT, AND PURE DEPHASING

Adiabaticity.– In order to evaluate the adiabaticity of our phase-driven system described by the Hamiltonian in Eq. (1) of the main text, we calculate the instantaneous adiabatic parameter for the dynamics confined to the two lowest energy levels. The instantaneous eigenstates of the abovementioned Hamiltonian are the well-known instantaneous number states

$$|n(t)\rangle = (2^n n! \sqrt{\pi} d_0)^{-\frac{1}{2}} \int d\varphi \exp\left[-\frac{1}{2}\left(\frac{\varphi - \varphi_d(t)}{d_0}\right)^2\right] H_n\left(\frac{\varphi - \varphi_d(t)}{d_0}\right), \tag{1}$$

where $d_0 = (2E_L/E_C)^{\frac{1}{4}}$ and H_n is the nth Hermite polynomial. By using known properties for the Hermite polynomials, we obtain two useful identities

$$\partial_t H_n\left(\frac{\varphi - \varphi_d(t)}{d_0}\right) = -\frac{\partial_t \varphi_d(t)}{d_0} \partial_n H_{n-1}\left(\frac{\varphi - \varphi_d(t)}{d_0}\right), \tag{2}$$

and

$$\frac{\varphi - \varphi_d(t)}{d_0} H_n\left(\frac{\varphi - \varphi_d(t)}{d_0}\right) = \frac{1}{2} H_{n+1}\left(\frac{\varphi - \varphi_d(t)}{d_0}\right) + n H_{n-1}\left(\frac{\varphi - \varphi_d(t)}{d_0}\right). \tag{3}$$

By taking a time-derivative of the instantaneous eigenstate in Eq. (1) and applying the identities given by Eqs. (2) and (3), we obtain

$$\langle m(t)|\partial_t|n(t)\rangle = \frac{\partial_t\varphi_d(t)}{\sqrt{2}d_0}\left(\sqrt{n+1}\delta_{m,n+1} - \sqrt{n}\delta_{m,n-1}\right), \tag{4}$$

where the remaining time-derivative depends on the details of the external drive protocol.

For the dynamics confined to the two lowest levels, the instantaneous adiabatic parameter is $\alpha_{\rm adi} = ||\hat{w}||/\omega_{10}$, where $||\hat{w}|| = \mathrm{Tr}\{\hat{w}^\dagger\hat{w}\}^{\frac{1}{2}}$ and $\hat{w} = -i\hat{D}_w^\dagger\partial_t\hat{D}_w$ generates the Berry connection. Here, $\hat{D}_w = |0(t)\rangle\langle 0_f| + |1(t)\rangle\langle 1_f|$ and $\{|0_f\rangle, |1_f\rangle\}$ is an orthonormal diabatic basis. By using Eq. (4), the adiabatic parameter takes a simple form

$$\alpha_{\rm adi} = \frac{|\partial_t\varphi_d(t)|}{\omega_{10}d_0} = \frac{\hbar|\partial_t\varphi_d(t)|}{(8E_CE_L^3)^{\frac{1}{4}}}. \tag{5}$$

For the resonant sinusoidal driving $\varphi_d(t) = \varphi_0 + \frac{2eV_0}{\hbar\omega_{10}}\sin(\omega_{10}t)$ used in the simulations in the main text, the adiabatic parameter becomes

$$\alpha_{rs} = \frac{2eV_0}{\hbar\omega_{10}d_0}|\cos(\omega_{10}t)| = \frac{2eV_0}{(8E_CE_L^3)^{\frac{1}{4}}}|\cos(\omega_{10}t)|. \tag{6}$$

In order for the master equation approach in the main text to be valid, Landau-Zener transitions must be suppressed at all times, such that any population transfer is dissipation–induced and occurs between instantaneous eigenstates. This is guaranteed by operating in the regime $\max(\alpha_{rs}) \ll 1$.

Quasiparticle-induced average shift of the system transition frequency.– The existence of quasiparticles induces an average frequency shift for the system that can be attributed to two different mechanisms [3, 4]: the quasiparticle renormalization of the Josephson energy and the quasiparticle-mediated virtual transitions between different energy levels. In general, each physical junction generates different shift terms, since nonvanishing total quasiparticle current requires conductance asymmetry (see previous section) and, hence, different superconducting gaps for the electrodes of the junctions ($\Delta_1 \neq \Delta_2$). However, our assumption of strong conductance asymmetry ($\Delta_1 \ll \Delta_2$) implies that the frequency shift is dominated by the junction with large quasiparticle flow, so we denote the effective gap by $\Delta \approx \Delta_1$. The renormalization term comprises of contributions from the pair tunneling and Josephson counterterms ($\hbar\omega_{10}, \delta E \ll 2\Delta$), as well as the terms in the quasiparticle tunneling Hamiltonian that do not contribute to pure dephasing and relaxation [4]. Since these contributions are renormalized Josephson energy terms for the individual junctions, they vanish for our effective junction. For a nonvanishing Josephson term, the quasiparticle contribution can be approximated knowing the CP-density-normalized quasiparticle density x_{qp} and the energy mode occupation of the quasiparticles at the gap $x_{qp}^A = f_{E,qp}(\Delta)$, where $f_{E,qp}$ is the energy mode of the lead quasiparticle distribution function [3].

By using the assumptions detailed in the main manuscript, the principal contribution from the virtual transitions to the ith energy level of the system is [3]

$$\delta E_{i,qp} = \sum_{k\neq i}\left|\langle k|\sin\frac{\hat{\varphi}}{2}|i\rangle\right|^2 F_{qp}(\omega_{ki}), \tag{7}$$

where $|i\rangle$ is the ith eigenstate with energy E_i, $\hbar\omega_{ki} = E_k - E_i$, and $F_{qp}(\omega)$ is an expression involving complex nested integration of the quasiparticle distribution in energy space [see Appendix A of Ref. [3]]. As it is apparent from Eq. (7), each energy shift generally accounts for virtual transitions to and from each other state in the Hilbert space. Our system lacks anharmonicity and, hence, we cannot restrict virtual occupation to the two lowest levels. However, we operate in the phase regime where $g_0 = [E_C/(32E_L)]^{1/4} \ll 1$ and, hence, Eqs. (4) and (5) in the main text imply that the largest terms in $\delta E_{i,\rm qp}$ correspond to virtual transitions between i and its energetically nearest levels. Thus, the frequency shift becomes

$$\hbar\delta\omega_{10,qp} = \delta E_{1,qp} - \delta E_{0,qp} = g_0^2\frac{1+\cos\varphi_d}{2}[F_{qp}(\omega_{10}) + F_{qp}(-\omega_{10})] + O(g_0^4), \tag{8}$$

where the non-nearest-neighbour terms are of the order g_0^4 by the constuction of the inner product in Eq. (7), whilst the nearest-neighbour terms scale as $e^{-g_0^2}g_0^2$, which was expanded to obtain the principal term in Eq. (8). Notably, the lowest-order contributions stem from the virtual transitions $1 \leftrightarrow 0$, $0 \leftrightarrow 1$, and $2 \leftrightarrow 1$. Making use of the definition

of quasiparticle impedance and assuming the high-frequency limit, we finally obtain

$$\hbar\delta\omega_{10,qp} = -g_0^2 \frac{\hbar g_T \Delta}{e^2} \left[x_{qp}\sqrt{\frac{2\Delta}{|\hbar\omega_{10}|}} - 2\pi x_{qp}^A \right] \frac{1+\cos\varphi_d}{2} + O(g_0^4), \tag{9}$$

where $g_T \propto G_{\text{eff}}$ is the effective junction conductance and e is the elementary charge. This term is generally time-dependent, due to the phase steering. We assume that the physical parameters are selected such that $\delta\omega_{10,qp} \ll \omega_{10}$, so that the frequency shift can be omitted. As evident from Eq. (9), the validity of this assumption is determined by the details of the execution of the low–energy limit given in the main text hindering it and the execution of the phase–regime as well as typical low quasiparticle densities supporting it. Note that in thermal equilibrium and at low temperatures $T \ll \Delta$, it is straightforward to support our statement that the frequency shift is dominated by the junction with large dissipative flow ($\Delta_1 \ll \Delta_2$) using Eq. (9), since then $\delta\omega_{10,qp} \propto e^{-\Delta/k_B T}$. This implies that $\delta\omega_{10,qp}^1/\delta\omega_{10,qp}^2 \propto e^{(\Delta_2-\Delta_1)/k_B T} \gg 1$, where the superscript indicates the physical junction.

Pure dephasing.– For our system, whose memristive operation relies solely on a weak but nonvanishing dissipative current generated by the quasiparticle–induced decay, pure dephasing adds another decoherence channel and, might potentially destroy the memristive behavior. Its effect would be to both distort the hysteresis loops by adding another time-dependent contribution to the quasiparticle current and to increase the memory-dependent damping of the average voltage by contributing to the total dephasing. Even though our SQUID is necessarily asymmetric as explained earlier, the individual junctions are assumed symmetric throughout this work. Thus, one would estimate pure dephasing using the self-consistent rate Γ_ϕ in Eq. (28) of Ref. [4] to avoid the issue of logarithmic divergence in the lowest-order perturbative tunneling treatment. However, the self-consistent rate scales with the system energy as $|A_s^d|^2$ where $A_s^d = (\langle 1|\sin\hat\varphi/2|1\rangle - \langle 0|\sin\hat\varphi/2|0\rangle)/2$ and the inner products can be calculated using the identities in Eqs. (10) and (11). This yields $|A_s^d|^2 = g_0^4 e^{-g_0^2}\sin^2(\varphi_d/2)/4$, which is of the order g_0^4, while the relaxation rate scales as g_0^2. A full comparison of the relaxation and pure dephasing rates would require knowledge of the quasiparticle distribution to calculate the quasiparticle spectral density in Eq. (3) of the main text, as well as to iteratively solve the self-consistent expression for the pure dephasing rate. As an example, assuming $\Gamma_\phi \ll \delta E$, the scaling becomes $\Gamma_\phi \propto |A_s^d|^2 \ln(1/|A_s^d|^2)$ [4], which decreases faster than $\Gamma_{1\to 0}$ in small g_0. In this work, we assume that the quasiparticle distribution can be established in a manner that the beneficial difference in scaling in the phase regime allows for the pure dephasing process to be neglected.

Finally, we remind the reader that our construction of the relaxation rate, as well as the considerations on the pure dephasing rate above, do not only exploit the adiabatic assumption but also that of low charateristic quasiparticle energies ($\hbar\omega_{10}, \delta E \ll 2\Delta$). This implies that the *secondary* terms in the rate equations in Ref. [4] can typically be omitted, since they are negligible in comparison to the primary terms we use throughout this work. However, our system crosses points during the phase-driving process in which the primary terms vanish and the secondary terms become the dominating contributions, ensuring that the total rates are nonvanishing. For example, the secondary term in the relaxation rate is proportional to $e^{-g_0^2}g_0^2(1-\cos\varphi_d)/2$, which reaches its maximum value at the point of vanishing primary term proportional to $e^{-g_0^2}g_0^2(1+\cos\varphi_d)/2$, that is, at $\varphi = \pi + 2\pi n$, $n \in \mathbb{Z}$. Even though the secondary terms dominate near these points, we assume that their contribution to the total dissipative flow during the full driving cycle is negligible due to the aforementioned assumptions. If low characteristic quasiparticle energies cannot be guaranteed, the full rate equations should be applied to study the potentially modified memristive function.

MATRIX ELEMENT OF $\sin\hat\varphi/2$ IN THE QUASIPARTICLE DECAY RATE

By using $\hat\varphi = 2g_0(\hat a + \hat a^\dagger) + \varphi_d(t)$, where $\hat a$ is the bosonic annihilation operator for the harmonic system, the sinusoidal phase term can be written as

$$\sin\frac{\hat\varphi}{2} = \frac{1}{2i}\left[e^{i\varphi_d/2}\hat D(ig_0) - e^{-i\varphi_d/2}\hat D(-ig_0)\right], \tag{10}$$

where $\hat D(ig_0) = \exp[ig_0(\hat a+\hat a^\dagger)]$ is the displacement operator with $ig_0 = i[E_C/(32E_L)]^{1/4}$ the phase space displacement. General properties for displaced number states assert that, for any pair of eigenstates of the harmonic oscillator $|n\rangle$ and $|m\rangle$, we have the identity [5]

$$\langle m|\hat D(\alpha)|n\rangle = \begin{cases} e^{-|\alpha|^2/2}\left(\frac{m!}{n!}\right)^{1/2}(-\alpha^*)^{n-m}\mathcal{L}_m^{n-m}(|\alpha|^2), & m \le n \\ e^{-|\alpha|^2/2}\left(\frac{n!}{m!}\right)^{1/2}\alpha^{m-n}\mathcal{L}_n^{m-n}(|\alpha|^2), & m \ge n, \end{cases} \tag{11}$$

where α is an arbitrary displacement and $\mathcal{L}_x^y$ denotes an associated Laguerre polynomial. The identity for $\alpha = ig_0$ results in

$$\langle m|\hat{D}(ig_0)|n\rangle \langle m|\hat{D}(-ig_0)|n\rangle^* = \langle m|\hat{D}(-ig_0)|n\rangle \langle m|\hat{D}(ig_0)|n\rangle^* = \begin{cases} (-1)^{n-m}|\langle m|\hat{D}(ig_0)|n\rangle|^2, & m \le n \\ (-1)^{m-n}|\langle m|\hat{D}(ig_0)|n\rangle|^2, & m \ge n. \end{cases} \tag{12}$$

Application of Eqs. (10) and (12) yields Eqs. (3) and (4) in the main text. It should be noted that a similar calculation has been performed in Ref. [3] for the pair $(|n\rangle, |0\rangle)$ and the results presented in the main text are a generalization to any pair of Fock states. The squared inner products corresponding to the transitions between the three lowest energy eigenstates are

$$\begin{aligned} |\langle 0|\sin\frac{\hat{\varphi}}{2}|1\rangle|^2 &= e^{-g_0^2}g_0^2\frac{1+\cos\varphi_d}{2}, \\ |\langle 0|\sin\frac{\hat{\varphi}}{2}|2\rangle|^2 &= \frac{1}{2}e^{-g_0^2}g_0^4\frac{1-\cos\varphi_d}{2}, \\ |\langle 1|\sin\frac{\hat{\varphi}}{2}|2\rangle|^2 &= \frac{1}{2}e^{-g_0^2}g_0^2(2-g_0^2)^2\frac{1+\cos\varphi_d}{2}. \end{aligned} \tag{13}$$

DERIVATION OF THE CHARGING, INDUCTIVE, AND QUASIPARTICLE CURRENTS

The average charging current for the junction is given by

$$\langle \hat{I}_{ch}\rangle = -2e\partial_t\langle \hat{n}\rangle = \frac{2e}{\hbar}E_L\mathrm{Tr}\{\hat{\rho}(\hat{\varphi}-\varphi_d)\} - 2e\mathrm{Tr}\{\hat{\mathcal{D}}\{\hat{\rho}\}\hat{n}\}, \tag{14}$$

where we assumed a Lindblad-form evolution $\partial_t\hat{\rho} = -i/\hbar[\hat{H}_S, \hat{\rho}] + \hat{\mathcal{D}}\{\hat{\rho}\}$ for the system density $\hat{\rho}$, and used the properties of the bosonic operators to rewrite the commutator $[\hat{n}, \hat{H}_S] = -i\hbar\omega_{10}(\hat{a}^\dagger + \hat{a})/(4g_0)$, after applying Ehrenfest theorem. By employing the operator for the current through the inductive element $\hat{I}_{ind} = -2e/\hbar E_L(\hat{\varphi} - \varphi_d)$, the average inductive current is

$$\langle \hat{I}_{ind}\rangle = \mathrm{Tr}\{\hat{\rho}\hat{I}_{ind}\} = -\frac{2e}{\hbar}E_L\mathrm{Tr}\{\hat{\rho}(\hat{\varphi}-\varphi_d)\}. \tag{15}$$

Hence, current conservation dictates that $\langle \hat{I}_{qp}\rangle = -\langle \hat{I}_{ch}\rangle - \langle \hat{I}_{ind}\rangle = 2e\mathrm{Tr}\{\hat{D}\{\hat{\rho}\}\hat{n}\}$. We assume the high-energy low-frequency regime discussed in the main text, so that $\hat{\mathcal{D}}\{\hat{\rho}\} = \hat{L}\hat{\rho}\hat{L}^\dagger - \{\hat{L}^\dagger\hat{L}, \hat{\rho}\}/2$, where $\hat{L} = \sqrt{\Gamma_{1\to 0}}\hat{a}$. Decomposing $\hat{\mathcal{D}}\{\hat{\rho}\}\hat{n}$ inside the trace and using the properties of $\hat{a}$ yields $\langle \hat{I}_{qp}\rangle = \Gamma_{1\to 0}(-e)\langle \hat{n}\rangle$, given in the main text.

DECAY OF HYSTERESIS WITH RESPECT TO THE SCALING OF T_1-TIMES

The same decay that enables hysteresis is responsible for the gradual decrease of the quasiparticle current due to dephasing. Observation of hysteresis requires a sufficently large T_1-time to be achieved. To estimate the bath-induced decay of the input and output values with respect to the scaling of the T_1-times, we present the change in the average quasiparticle current after 10 consecutive driving cycles in Fig. 1. Since the system is initialized to the classical trajectory, $\langle \hat{I}_{qp}\rangle|_{t=0}$ corresponds to the value that the classical memristor returns after each driving cycle. Note that the memory-dependent damping in the input $\langle \hat{V}\rangle$ determines the decay and, hence, has the same scaling as the output. As briefly mentioned in the main text, the magnitude-scaled parametric hysteresis curves do not noticeably decay after 10 cycles for the orders of magnitude of the T_1-times between 1 ms and 10 μs. This is noticeable in Fig. 1, where the quasiparticle current after 10 cycles does not return to its original value when $\log[S_{qp}(\omega_{10})/\omega_{10}] > -2$.

EFFECT OF INITIALIZATION ON THE HYSTERETIC BEHAVIOR AND MEMORY QUANTIFIER

Here, we provide a qualitative interpretation for the varied hysteretic curves displayed in Fig. 3 of the main text. As in Fig. 2 of the main text, initially assuming a nonzero voltage over the SQUID and a negligible inductive phase difference yields hysteresis with π-rotational symmetry in the current-voltage plane (blue curve). On the other hand, assuming vanishing initial voltage and nonzero inductive phase difference destroys the hysteretic behavior (red curve).

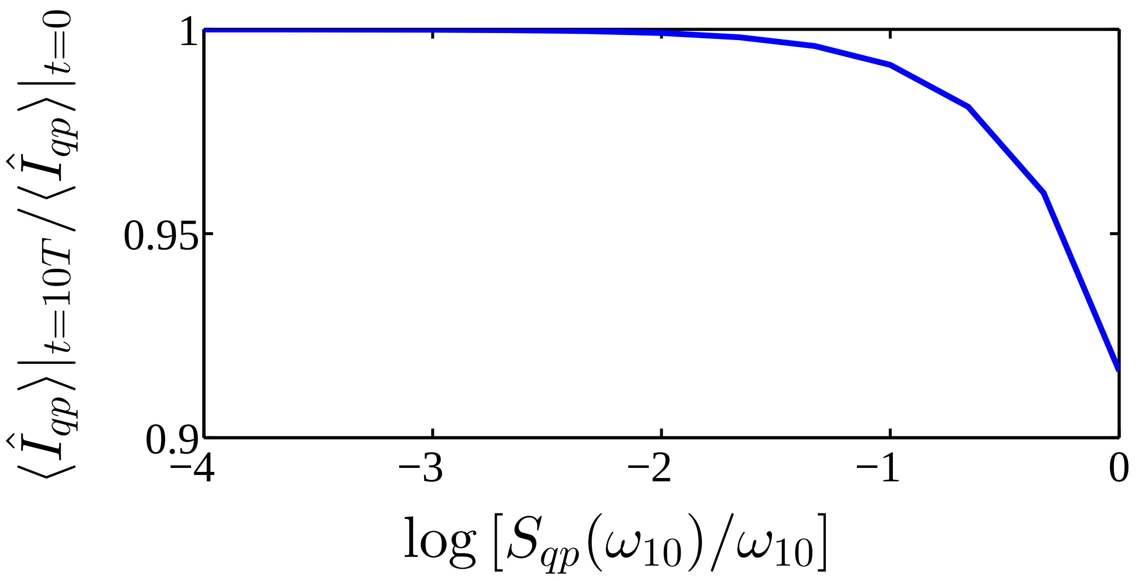

FIG. 1. Expectation value of the quasiparticle current after 10 resonant sinusoidal driving cycles with respect to the spectral density of the quasiparticle bath. System parameters and initialization as in Fig. 2 of the main text.

Finally, assuming that both are nonvanishing allows for voltage-asymmetric hysteresis (black curve). Each case can be seen as an example of the time-symmetry of the quasiparticle current between two consecutive crossings of the zero-energy point. With sinusoidal driving, both the voltage and the memristance are time symmetric, whereas the symmetry of their product, the quasiparticle current, depends on the specific initialization. Hence, tuning the initial conditions yields a variety of different memristic switchings which can, in general, be made voltage asymmetric. The parametric current-voltage characteristics can also be tuned by resorting to different bias protocols which may help to achieve desired behavior.

The memory quantifier for Fig. 2 in the main text decays pairwise linearly from $N_m^1/N_m^{cl} = 0.9960$ to $N_m^{19}/N_m^{cl} = 0.8511$, where N_m^{cl} is the value for the classically initialized weak-damping solution, during the simulation. In the weak-damping regime, the decay is negligible. For the different initializations in Fig. 3 of the manuscript, the weak-damping solutions yield $N_m/N_m^{cl} = 1$ (blue), $N_m/N_m^{cl} \propto 10^{-7}$ (red), and $N_m/N_m^{cl} = 1.3409$ (black). Curiously the last case yields the same value for both asymmetric loops.

AREA OF A HYSTERESIS LOOP AS A MEMORY MEASUREMENT

For a classical voltage-controlled memristive system, the current response is generally given by [6]

$$I(t) = G(\gamma(t), V(t), t)V(t), \tag{16}$$

where $G(\gamma, V(t), t)$ is the memductance, which depends on the instantaneous value of the input voltage $V(t)$, the accumulated value of the memory variable $\gamma(t)$, and the time t in a parametric manner. The memory variable update is defined by

$$\partial_t \gamma(t) = f(\gamma(t), V(t), t), \tag{17}$$

where the update function f depends on the past evolution, the input and any external parametric driving. Notice that the general definitions used above allow for tracking a specific state variable γ rather than absorbing the total response into a generalized memory variable γ_x such that $G(\gamma_x) = G(\gamma, V, t)$ where $\gamma_x = f_x(\gamma_x, V, t)$ now updates the new variable. This absorption can always be done, since memductance is a system property depending on the input and external driving via the state variable.

Define $\mathcal{C}$ as any closed curve in the (I, V)-space, hence the area enclosed by the curve is determined by Green's

theorem as

$$A = \frac{1}{2}\oint_{\mathcal{C}}(VdI - IdV) = \frac{1}{2}\oint_{\mathcal{C}} V^2 d\left(\frac{I}{V}\right) = \frac{1}{2}\oint_{\mathcal{C}} V^2 dG, \tag{18}$$

where the explicit dependences given by the response in Eq. (16) are omitted for clarity. Transforming to temporal space, the integration takes the form

$$A = \frac{1}{2}\int_0^T dtV^2(\partial_\gamma G \partial_t \gamma + \partial_V G \partial_t V + \partial_t G), \tag{19}$$

where we have fixed the loop to begin at time $t = 0$ and end at $t = T$ when the initial point in the (I, V)-space is reached again. Assuming pinched hysteresis, the periodicity condition is conveniently written as $V(nT) = 0$, $n \in \mathbb{Z}$. Due to this condition, the second term in Eq. (19) yields

$$\frac{1}{2}\int_0^T dtV^2 \partial_V G \partial_t V = \frac{1}{2}\int_{V(0)}^{V(T)} dVV^2 \partial_V G = 0, \tag{20}$$

implying that any instantaneous voltage dependence in the current response does not contribute to the area. By making use of Eq. (17), the area is

$$A = \frac{1}{2}\int_0^T dtV(t)^2[\partial_\gamma G(\gamma, V, t) f(\gamma, V, t) + \partial_t G(\gamma, V, t)], \tag{21}$$

where the first term in the integrand corresponds to the response related to the selected memory variable and the second term accounts for any remaining explicit time dependence of the conductance.

Using the notation above, any non-linear conductor $G = G(V)$ can be defined via $\gamma = V$ such that $f(\gamma) = \partial_t V$. Equation (21) then yields

$$A = \frac{1}{2}\int_0^T dtV(t)^2 \partial_V G(V) \partial_t V = \frac{1}{2}\int_{V(0)}^{V(T)} dVV^2 \partial_V G(V) = 0, \tag{22}$$

where the last equality is due to the periodicity. In other words, purely non-linear conductance does not produce hysteresis, and cannot generate a non-zero loop area. The memory-variable related term in Eq. (21) can be rewritten as

$$\begin{aligned} \frac{1}{2}\int_0^T dtV(t)^2 \partial_\gamma G(\gamma, V, t) f(\gamma, V, t) &= \frac{1}{2}\left[\int_0^T V(t)^2 \partial_\gamma G(\gamma, V, t) \int_0^T dt f(\gamma, V, t) \right. \\ &\quad \left. - \int_0^T dt \int_0^t d\tau f(\gamma, V, \tau) \partial_t [V(t)^2 \partial_\gamma G(\gamma, V, t)]\right] \\ &= -\frac{1}{2}\int_0^T dt \Delta\gamma(t) \partial_t [V(t)^2 \partial_\gamma G(\gamma, V, t)], \end{aligned} \tag{23}$$

where $\Delta\gamma(t) = \gamma(t) - \gamma(0)$ is the change in the memory variable from its initial value, we executed integration by parts after the first equality, and the first term after the first equality vanishes due to the periodicity condition. Applying the results in Eqs. (21) and (23) yields the definition of the loop area as a memory quantifier given in the main text for our superconducting circuit.

[1] Peotta, S. & Di Ventra, M. Superconducting memristors. *Phys. Rev. Applied* **2**, 034011 (2014).

[2] Schwabl, F. *Quantum Mechanics, 4th ed.* (Springer, New York, 2007).

[3] Catelani, G., Schoelkopf, R. J., Devoret, M. H. & Glazman, L. I. Relaxation and frequency shifts induced by quasiparticles in superconducting qubits. *Phys. Rev. B* **84**, 064517 (2011).

[4] Catelani, G., Nigg, S. E. , Girvin, S. M., Schoelkopf, R. J. & Glazman, L. I. Decoherence of superconducting qubits caused by quasiparticle tunneling. *Phys. Rev. B* **86**, 184514 (2012).

[5] Hollenhorst, J. N. Quantum limits on resonant-mass gravitational-radiation detectors. *Phys. Rev. D* **19**, 1669 (1979).
[6] Di Ventra, M., Pershin, Y. V. & Chua, L. O. Circuit elements with memory: Memristors, memcapacitors, and meminductors. *Proceedings of the IEEE* **97**, 1717 (2009).
